# Pediatric melanoma—The whole (conflicts of interest) story^☆^

**DOI:** 10.1016/j.ijwd.2018.10.020

**Published:** 2018-11-19

**Authors:** Klaus Rose, Jane M. Grant-Kels

**Affiliations:** a klausrose Consulting, Riehen, Switzerland; b University of Connecticut Health Center, Farmington, Connecticut

**Keywords:** Pediatric melanoma, Pediatric legislation, Pediatric investigation plan, FDA pediatric written request, Pediatric clinical pharmacology, Developmental pharmacology

## Introduction

Pediatric melanoma, although rare, exists. True childhoood, pre-adolescent melanomas are different from conventional adolescent and adult melanomas (Pappo, 2014, Rose and Grant-Kels, 2018a). Since 2011, an ever-increasting number of new, approved, and efficacious drugs, drug combinations, and innovative treatments are under clinical investigation, including re-programming immune cells that attack and defeat malignant melanoma cells (Geukes Foppen et al., 2015, Goff et al., 2016, Rosenberg et al., 2011).

Despite these innovations, some academic careers are being built on pediatric studies in melanoma and other diseases that provide limited or no scientific contribution. The U.S. Food and Drug Administration (FDA), under the claim of improving child health care, is on a mission against pediatric off-label use in close collaboration with the American Academy of Pediatrics (AAP) on the basis of the concept that children are therapeutic orphans and discriminated against in drug treatment and development (Ward et al., 2018). The European Medicines Agency (EMA) has further expanded on this quest.

The FDA and EMA define children administratively rather than physiologically: age ≤ 16 years (FDA) (Hirschfeld, 2012) and < 18 years (EMA) (Hirschfeld and Saint-Raymond, 2011). The EMA's condemnation of pediatric off-label use as always dangerous (EMA, 2004) is counter to general practice and experience. Today's system of clinical studies as the basis for drug approval evolved as an aftermath of society's efforts to balance therapeutic promises of innovative drugs against their potential downsides following the thalidomide catastrophe (Rägo and Santoso, 2008, Thomann, 2007, Vargesson, 2015). Subsequently, the FDA required separate drug approval and separate studies in children, as if children were a different species (Karesh, 2015). Herein we challenge this approach.

Toxicities were reported in newborns in the early days of antibiotic treatments (American Academy of Pediatrics, Committee on Drugs, 1995, Burns et al., 1959, Silverman, 1956). Resulting alarmistic warnings with regard to such toxicities were and are used to stoke fears in mothers, families, physicians, and health care professionals and to suggest that children might be harmed by drugs not specifically tested in children. This overlooks that children mature and do not remain neonates. Their organs develop and mature rapidly over months. Instead of applying the learnings of developmental pharmacology (Kearns et al., 2003) to drug approval and clinical practice, fears are amplified and used to justify questionable, label-focused, regulatory studies performed by clinicians (Turner et al., 2017) and coordinated by the FDA and EMA (Dunne et al., 2012, Saint-Raymond and Brasseur, 2005, Tomasi et al., 2017). These studies are paid for initially by pharmaceutical companies (Li et al., 2007, Rose and Grant-Kels, 2018b) but ultimately by patients, their families, and the tax payers. The highest price is paid by the young patients who are prohibited from already approved efficacious safe therapies because of these misconceptions.

Pediatric melanoma remains a paradigm of profound and unsolved challenges of innovative medicine because of the 1) contrast between how well young patients with melanoma could be treated versus how they are not given access to some of these new medications and are enrolled in questionable studies; 2) kafkaesque interference in medical treatment by the FDA and EMA; and 3) grudging and partial recognition by regulators and clinicians of committed errors in this arena (Geoerger et al., 2017, Rose and Grant-Kels, 2018b) To ethically and successfully advise and treat young patients with melanoma and their families, physicians need an intellectual compass to navigate the jungle of conflicting information, false promises, and useless studies (Rose and Grant-Kels, 2018b).

## Historical background

Historically, there have been only a few effective systemic drugs available: opioids, alcohol, hashish, and poisons. Over time, medicine and society have become more sophisticated and complex, resulting in new laws and the establishment of institutions to oversee production, commerce, and the use of drugs. The U.S. Food and Drug Act of 1906 prohibited the interstate transport of adulterated food and drugs (Janssen, 2008). Initially, the Bureau of Chemistry was in control, but as of 1930, the FDA has assumed this responsibility. At the onset, medication labels described the content of a product, but over the following decades, this has evolved into describing the content and medical use(s). Other new institutions, including the American Boards of Internal Medicine and Medical Specialties, the state licensing authorities, and the American Medical Association, have become involved in the governing and licensing of U.S. physicians (Hamowy, 1979).

World War II brought both horror and innovations, including the industrial production of antibiotic treatments (Hilts, 2003). The postwar euphoria of drug development expansion was shattered when, in 1961, a sedative treatment that was licensed for over-the-counter use by the German authorities was unveiled to have caused severe malformations in thousands of newborns worldwide (Thomann, 2007, Vargesson, 2015). The resultant public uproar precipitated the 1962 U.S. law that required proof of efficacy and safety of drugs by appropriate studies before FDA approval (Hirschfeld and Saint-Raymond, 2011, Ward et al., 2018).

Although accepted worldwide today (Rägo and Santoso, 2008), this principle broke taboos in 1962 and replaced the verdict of eminent experts with anonymous, FDA-controlled data (Hilts, 2003). The same law also transferred the oversight of drug advertising to the FDA (Donohue, 2006). In the 1950s, acute toxicities in preterm newborns from antibiotic treatments became publicized (American Academy of Pediatrics, Committee on Drugs, 1995, Burns et al., 1959, Silverman, 1956). The FDA's new oversight over advertising caused lawyers to recommend pediatric warnings in drug labels to avoid damage lawsuits. Dr. Harry C. Shirkey, the first chairman of the AAP committee on drugs, interpreted these warnings medically and concluded that children were "therapeutic orphans" to whom many modern drugs were denied (Shirkey, 1968). The AAP subsequently established a close collaboration with the FDA (Ward et al., 2018) and complained in 1977 that many drugs could not be advertised for children (American Academy of Pediatrics, 1977).

In 1979, the FDA defined children as individuals age ≤ 16 years (Hirschfeld, 2012). In 1995, the AAP demanded separate pediatric drug studies (American Academy of Pediatrics, 1995). Since 1997, the United States rewards pediatric studies that are sponsored by pharmaceutical companies and execute an FDA written request with a 6-month patent extension (Li et al., 2007, Hirschfeld and Saint-Raymond, 2011). Written request studies are voluntary, but as of 2003, a second law authorized the FDA to demand pediatric studies without reward (Hirschfeld and Saint-Raymond, 2011). As of 2012, pediatric laws are no longer time limited (Ward et al., 2018). In 2007, the European Union pediatric regulation came into force and demanded pediatric investigation plans (PIPs) for new drugs (Hirschfeld and Saint-Raymond, 2011, European Union, 2006). The European Union defines children as those age < 18 years and has fewer limitations than U.S. laws. PIPs are also demanded for rare diseases, vaccines, and biologic treatments. So far, more than 1000 PIPs have been issued (European Union, 2017), with each demanding one, few, or many pediatric studies. FDA-requested/demanded and PIP-demanded pediatric studies ask predominantly for separate proof of efficacy in minors (Field and Boat, 2012, Rose and Grant-Kels, 2018b) and often include young adults age ≤ 21 years. Many patients in these studies are physiologically no longer children but rather adolescents and young adults.

## Medication and children

Neither the AAP nor the FDA have ever defined children physiologically vis-á-vis drug treatment, and the same holds true for AAP's definition. To define the age of patients who should be cared for by pediatricians is appropriate (Hardin and Hackell, 2017), but not for drug treatment. Although there are strong desires to support and protect children (e.g., Declaration of the Rights of the Child [Unicef, 2003] and Convention on the Rights of the Child [United Nations, 1996]), this goal is prone to abuse.

When Dr. Shirkey characterized children as “therapeutic orphans,” he established a blur at the interface of medicine and law (Rose and Walson, 2017b). The legal definition of children is chronological, but no physiological switch transforms adolescents into adults in the night of their 16^th^ or 18^th^ birthday. The separation of pediatric versus adult populations for pharmaceutical treatment is physiologically flawed, except perhaps in the neonatal period. Neonates' absorption, distribution, metabolism, and excretion (ADME) differ from adults’, and typical neonatal toxicities exist (e.g., grey baby syndrome secondary to chloramphenicole). Chloramphenicole toxicities occur rarely in older children, but they are different from neonatal toxicities (Wiest et al., 2012).

In the 1950s, the measurement of pediatric ADME barely existed but does exist today (Kearns et al., 2003). The U.S. Pediatric Pharmacy Advocacy Group was established in 1979 (Poole, 2010) and the European Society for Developmental Perinatal and Paediatric Pharmacology in 1988 (European Society, 2013). Despite this, representatives in developmental pharmacology claimed "CONTINUED PEDIATRIC THERAPEUTIC DISASTERS [sic]” still in 1999 (Christensen et al., 1999) when the lessons of neonatal toxicities had already transformed neonatology. The listed "disasters" occured exclusively in neonates (Christensen et al., 1999), but such warnings were a good marketing pitch that extrapolated neonatal toxicities for all children and resulted in the demand for separate studies.

## Different clinical studies

The first systematic pediatric studies occured in pediatric oncology (Adamson, 2015). Pioneers tested chemotherapy and were rewarded with unexpected survival rates. Two major factors were absent: These successes were not achieved by new drugs but by well-known cytotoxic treatments (Adamson, 2015), and the studies were not regulatory studies.

The pediatric oncology studies that were rewarded by the FDA as of 1997 on were different and investigated individual cytotoxic treatments mostly in terminally ill young patients (U.S. Food and Drug Administration, 2001b, U.S. Food and Drug Administration, 2003, Fraser et al., 2014, Geoerger et al., 2012, Wharton et al., 2014). However, at this time, pediatric oncology no longer routinely used monotherapy, and instead used complex treatment protocols. For example, one clofarabine study in acute myeloblastic leukemia was performed from 2002 to 2004 (phase 2 study of clofarabine). In 2004, the FDA approved clofarabine for relapsing/remitting acute lymphablastic leukemia in pediatric patients ages 1 to 21 years after treatment failure with two prior regimens (FDA clofarabine label; Jeha et al., 2006). However, the label emphasizes that no trials show an improvement in disease-related symptoms or increased survival (U.S. Food and Drug Administration, 2015).

Clinically, a separate pediatric approval of cytostatic treatments is irrelevant. FDA-rewarded pediatric oncology studies were regulatory studies with a focus on labels, not on patients. Children (i.e., older than neonates) need drugs in the right doses and combinations but respond to medications in the same way as adults without the need for efficacy studies in their age group before they can gain access to newer, more efficacious therapies. The conflict is between the FDA's vision of labels as instructions for physicians and physicians' right to treat their patients.

Another example is nab-paclitaxel in patients age 6 months to 17 years with relapsing/remitting solid tumors. The report omits that the study was demanded by PIP EMEA-001308-PIP01-12-M01 (Moreno et al., 2018), and Celgene did not sponsor this study voluntarily. For patients and parents, this study offered false hope because it was a regulatory study with 58% of patients age 12 to 17 years, and adolescents/young adults do not need separate dose findings.

## Melanoma

Today, pediatric melanoma is differentiated into conventional melanoma, true childhood melanoma that arises prior to puberty, Spitzoid melanoma, and melanoma that arises in giant congenital melanocytic nevi. Conventional melanomas in adults and adolescents are genomically similar enough to be treated the same (Pappo, 2014, Rose and Grant-Kels, 2018a). The classification of melanoma in legally minor patients as pediatric is regulatory, but medically inappropriate. A chronological classification is currently used worldwide, which results in many pediatric melanoma publications that list and discuss conventional melanomas in legally underage patients (Bartenstein et al., 2018, Brecht et al., 2015, Eggen et al., 2018, Offenmueller et al., 2017). In the past, the age limit of pediatric melanoma was clinically irrelevant, but this is rapidly changing with the increasing number of treatments. A classification of legally underage patients as children can have fatal life-or-death consequences if medications that are potentially efficacious are withheld.

Ipilimumab was one of the first FDA-registered anti-melanoma compounds. The FDA ipilimumab melanoma written request requested four studies, including dose escalation in pediatric patients ages 1 to 21 years with refractory cancers and pharmacokinetic and safety in pediatric patients ages 1 to ≤ 18 years with unresectable or metastatic melanoma (U.S. Food and Drug Administration, 2014). The written request was preceded by a pediatric ipilimumab National Cancer Institute study that had started in 2008 (phase 1 study of ipilimumab).

Originally, the EMA had issued a class waiver for pediatric melanoma but withdrew adolescent melanoma from the list of PIP-exempted diseases in 2008 (Rose and Walson, 2017a). Since then, 13 PIPs have been issued, of which 12 (including the original ipilimumab melanoma PIP) demand systemic monotherapy studies in melanoma or solid tumors (including melanoma) (European Medicines Agency, 2011a, Rose and Grant-Kels, 2018a). One PIP demands the local injection of talimogene into melanoma and other noncentral nervous system malignant solid tumors (European Medicines Agency, 2001). The first two ipilimumab PIP studies correspond to the FDA-requested studies (European Medicines Agency, 2011). The National Cancer Institute study started in 2008, and obviously the manufacturer negotiated with the FDA and EMA for a written request and PIP with overlapping studies.

The ipilimumab written request/PIP-requested pediatric dose-finding study was reported in 2016 (Merchant et al., 2016) and the pharmacokinetic and safety study in 2017 (Geoerger et al., 2017). The dose-finding study recruited 33 patients, including 12 patients with melanoma. The study report does not give the age of the patients with melanoma (Merchant et al., 2016). The pharmacokinetic and safety study was terminated with 14 enrolled patients, of whom 12 were treated with ipilimumab (U.S. Food and Drug Administration, 2017, Geoerger et al., 2017, Rose and Grant-Kels, 2018a, Rose and Grant-Kels, 2018b). Recruitment had waned because the superiority of ipilimumab plus nivolumab had resulted in FDA approval (FDA clinical review ipilimumab, 2017). The FDA approved ipilimumab in 2011, but the National Cancer Institute pediatric study continued (Merchant et al., 2016). The study was not pediatric and should have been stopped in 2011 for patients age ≥ 18 years. A PIP-demanded phase 1 pediatric melanoma study with vemurafenib in adolescents was terminated because recruitment had waned (Rose and Grant-Kels, 2018b, Rose and Walson, 2017a).

The pediatric ipilimumab dose-finding study in patients age ≤ 21 years was unethical. Dose finding in young patients is legitimate but medically senseless in adolescents with a mature body and borders on criminal in legal adults age 18 to 21 years. We doubt that the omission of the melanoma patients’ age was by chance (Merchant et al., 2016), and the corresponding author's email is no longer functional. Also, the two terminated pediatric melanoma studies were unethical from the beginning. Regulatorily, they were pediatric studies, but medically they were not.

The EMA silently changed the vemurafenib PIP (European Medicines Agency, 2010) into a waiver (no pediatric studies demanded; current vemurafenib PIP). The ipilimumab PIP, now in its seventh modification, still lists the dose escalation (Merchant et al., 2016) and pharmacokinetic and safety studies (Geoerger et al., 2017) that were demanded in the original ipilimumab PIP (European Medicines Agency, 2011b). An efficacy, safety, and tolerability study that was demanded in the original PIP for the comparison of adjuvant ipilimumab with high-dose interferon α-2b in children age 12 to ≤ 18 years (and adults) with resected high-risk melanoma is now listed as "Deleted in procedure EMEA-000117-PIP02-10-M07" (European Medicines Agency, 2017).

Melanoma PIPs require pharmacokinetic data in pediatric patients age ≤ 17 years and some also in young adults (Rose and Walson, 2017a). Talimogene is an oncolytic for injection into unresectable melanoma tumors (Fountzilas et al., 2017), and its PIP demands two injection studies into melanoma tissue or other advanced noncentral nervous system tumors in pediatric patients age 2 to 17 years (European Medicines Agency, 2001). Five industry-sponsored PIP-demanded studies with pembrolizumab, dabrafenib, paclitaxel, cobimetinib, and talimogene laherparepvec in children, adolescents, and young adults with melanoma and other tumors are currently recruiting worldwide (Rose and Grant-Kels, 2018b).

The intention of the melanoma written request and PIPs is not improved clinical care but labels. The chronological definition of children, combined with the European Union's lack of limitations, have led to a worldwide epidemic of PIP-demanded questionable pediatric studies (Rose, 2014, Rose and Grant-Kels, 2018c, Rose and Grant-Kels, 2018d, Rose and Grant-Kels, 2018e, Rose and Happle, 2017, Rose and Kopp, 2015, Rose and Müller, 2016; Rose and Senn, 2014, Rose and Walson, 2015, Rose and Walson, 2017a), incorporating studies in solid tumors including melanoma (Rose and Grant-Kels, 2018a, Rose and Grant-Kels, 2018b, Rose and Walson, 2017b). The pediatric melanoma studies compete worldwide for rare patients. The EMA epidemiological assumptions equalize the number of diagnosed melanomas in young patients with the number of patients that require systemic treatment, which is incorrect for the majority where the tumor is excised (Rose and Senn, 2014).

PIP negotiations will continue unless this scenario is addressed and discontinued. There are conflicts of interest and institutional inertia. The publication of the phase 2 ipilimumab study recommends that in the future, young patients should participate in promising pivotal cancer studies (Geoerger et al., 2017). Some, but not all, authors seem to be aware that this study was questionable. The recommendation might be a compromise between authors aware of the genomic similarity of conventional melanoma in younger and older patients (Pappo, 2014) and others, for whom EMA-triggered pseudoscientific pediatric studies are more important (Geoerger et al., 2012, Geoerger et al., 2017).

## Discussion

Institutions with too much power enforce senseless rules and regulations. The preamble of the European Union pediatric regulation is a harangue against the market (European Union, 2006). Nonetheless, the market (and not FDA/EMA-triggered regulatory pseudoscientific studies) offers new hope to young patients with acute lymphoblastic leukemia (Maude et al., 2018).

Early pediatric oncology focused on patients. Slowly, FDA administrative power grew, and the term "off-label" emerged in 1988 (Plate, 2009). The FDA definition of children as those age ≤ 16 years conveyed an inappropriate physiologic connotation to this age limit. The U.S. law of 1906 established a balance between the jurisdictions of the medical profession and FDA-control of interstate drug commerce. The AAP has always pragmatically defended pediatric off-label use (American Academy of Pediatrics, 2002) but played a crucial role in the emerging separate pediatric drug approval. Parents of children with cancer must sign informed consent acknowledging that the drugs used are not licensed. Although neonatology, developmental pharmacology, and pediatric oncology were already mature subdisciplines in 1997, FDA-rewarded separate pediatric studies for many diseases in such heterogenous populations made them scientifically worthless. Pediatric antidiabetic, antihypertensive, antidepressant, and other studies recruited too diversely aged patients. Drugs do not work differently one day before or after one‘s 17^th^ or 18^th^ birthday.

Most diseases are rare in minors, and most FDA-rewarded pediatric studies recruit(ed) internationally (Dunne et al., 2012, Pasquali et al., 2010). Furthermore, the pediatric population was often expanded to young adults ≤ 21 years (Merchant et al., 2016). The European Union has further expanded this approach by demanding pediatric studies for virtually any new drug and constantly removes diseases from its list of class waivers. Since 2018, the EU has demanded pediatric studies for liver cancer and Parkinson's disease (European Medicine Agency, 2015). Except for justified demands for pediatric formulations and broadened EU public acceptance for pediatric research, probably the best outcome of the EU PIPs is that their exaggerations have helped to unveil the flaws already dormant in the U.S.-born concept of children as therapeutic orphans.

Herein, we review that the regulatory authorities of the world's most advanced regions provide funds for questionable studies. The FDA, EMA, and researchers carefully codify their wording and claim clinical concerns for children, but use regulatory endpoints for results. Until approximately 2000, many believed that more pediatric studies would improve child health care. The 2001 FDA Report to Congress mused about potential clinical outcomes of pediatric exclusivity (U.S. Food and Drug Administration, 2001a). In the 2016 report, the clinical outcomes were skipped and replaced with regulatory endpoints (U.S. Food and Drug Administration, 2016b). The FDA allows for the extrapolation of efficacy in some clinical areas (Dunne et al., 2011, Sun et al., 2017).

Experienced physicians and pediatricians have always used common sense and prescribed medications off-label. Even Dr. Shirkey noted that most physicians simply ignored the pediatric warnings (Shirkey, 1968). The FDA is not monolithic. In some areas, the FDA no longer insists on separate pediatric proof of efficacy, including dermatology (U.S. Food and Drug Administration, 2016a) and epilepsy (Sun et al., 2017).

## Conclusions

In our opinion, we have reviewed the largest systematic abuse of patients in medical research in history, dwarfing the scale of unethical studies unveiled in 1966 (Beecher, 1966). All written request/PIP-triggered studies are performed in centers that are committed to the highest ethical standards. Pediatric studies are approved by institutional review boards/ethics committees and regulatory authorities. The Declaration of Helsinki states that medical research should help to understand the causes, development, and effects of diseases and to improve preventive, diagnostic, and therapeutic interventions (World Medical Association).

Most FDA/EMA requested/demanded pediatric studies are in open breach of the Declaration of Helsinki. Too many academic careers have been built on questionable activism. Safety mechanisms to protect patients in medical research are in place worldwide, but they work only if institutional review boards/ethics committees identify such studies as questionable. Questionable studies are justified in appearingly scientific disguise. Worldwide, institutional review boards/ethics committees will need training on U.S./European Union pediatric legislation. With the internet, written requests and PIPs that financially drive questionable pediatric studies are easy to find. Furthermore, www.clinicaltrials.gov allows for the identification of multiple PIP-driven studies in diseases that are rare in young patients, including melanoma, leukemia, multiple slerosis, and psoriasis (Rose and Happle, 2017, Rose and Kopp, 2015, Rose and Müller, 2016, Rose and Walson, 2015). Institutional review boards/ethics committees should suspend questionable pediatric studies and reject new ones.

Pediatric research needs to be addressed by the World Medical Association, and a separation of reasonable from FDA/EMA-demanded studies should be established. Compliance with the Recommendations for the Conduct, Reporting, Editing, and Publication of Scholarly Work in Medical Journals is required today by most leading biomedical journals (International Committee, 2017). They do not address conflicts of interest of regulatory authorities and some pediatric researchers. They need an update to prevent authors from regulatory authorities from not revealing their conflicts of interest (Mentzer, 2014, Saint-Raymond and Brasseur, 2005, Tomasi et al., 2017, Tsukamoto et al., 2016, Wharton et al., 2014) and pediatric researchers from omitting FDA/EMA decisions that trigger payment by companies (Casanova et al., 2016, Falkner, 2017, Fraser et al., 2014, Gaspar et al., 2018, Geoerger et al., 2012, Geoerger et al., 2017, Hoppu et al., 2012, Jeha et al., 2006, Merchant et al., 2016, Moreno et al., 2018, Pearson et al., 2017, Ruperto et al., 2013, Siegfried et al., 2018, Snyder et al., 2013, Torok et al., 2018, Turner et al., 2014, Turner et al., 2017, Ward et al., 2018).

Some campaign for a further expansion of FDA-mandated studies and PIPs (U.S. Food and Drug Administration, 2016b, European Medicines Agency, 2016). Physicians need to learn about regulatory affairs and differentiate label-focused senselessness from well-intended reasonable studies. There is no scientific reason to separate drug registration in adults and children. Where there are contraindications, such as for chloramphenicol in neonates, they should be incorporated into the labels. Otherwise, the current system should be replaced with dose recommendations for adults and children in both the United States and European Union. Questionable pediatric studies in solid tumors, including melanoma, should be suspended.
